# 2D Hybrid Perovskite Sensors for Environmental and Healthcare Monitoring

**DOI:** 10.1021/acsami.4c02966

**Published:** 2024-06-05

**Authors:** Karl Jonas Riisnaes, Mohammed Alshehri, Ioannis Leontis, Rosanna Mastria, Hoi Tung Lam, Luisa De Marco, Annalisa Coriolano, Monica Felicia Craciun, Saverio Russo

**Affiliations:** † Centre for Graphene Science, College of Engineering, Mathematics and Physical Sciences, University of Exeter, Exeter EX4 4QL, U.K.; ‡ Institute of Nanotechnology, Via Monteroni, Lecce 73100, Italy

**Keywords:** perovskites, photodetector, 2D materials, encapsulation, environmental sensing, photoplethysmography, beeswax

## Abstract

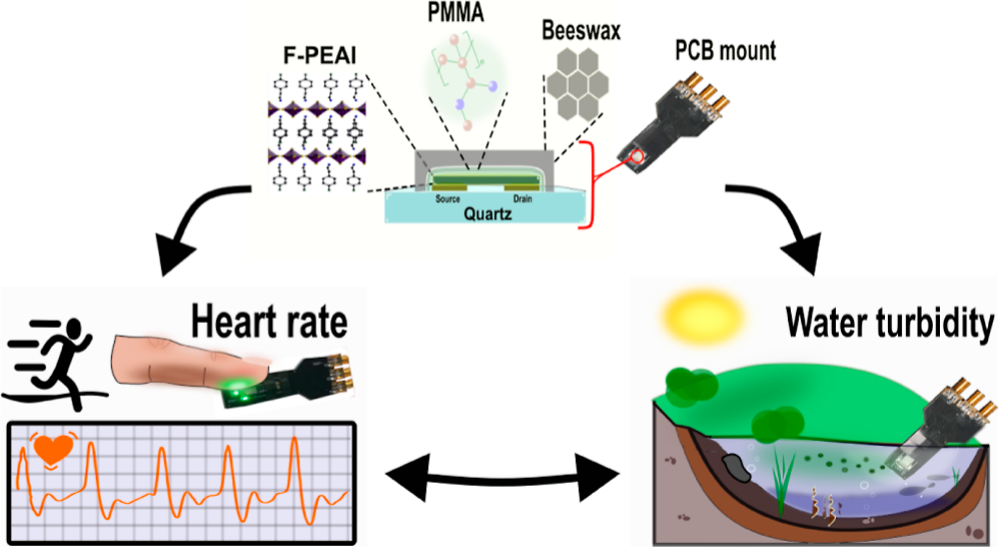

Layered perovskites, a novel class of two-dimensional (2D) layered materials, exhibit versatile photophysical properties of great interest in photovoltaics and optoelectronics. However, their instability to environmental factors, particularly water, has limited their utility. In this study, we introduce an innovative solution to the problem by leveraging the unique properties of natural beeswax as a protective coating of 2D-fluorinated phenylethylammonium lead iodide perovskite. These photodetectors show outstanding figures of merit, such as a responsivity of >2200 A/W and a detectivity of 2.4 × 10^18^ Jones. The hydrophobic nature of beeswax endows the 2D perovskite sensors with an unprecedented resilience to prolonged immersion in contaminated water, and it increases the lifespan of devices to a period longer than one year. At the same time, the biocompatibility of the beeswax and its self-cleaning properties make it possible to use the very same turbidity sensors for healthcare in photoplethysmography and monitor the human heartbeat with clear systolic and diastolic signatures. Beeswax-enabled multipurpose optoelectronics paves the way to sustainable electronics by ultimately reducing the need for multiple components.

## Introduction

Layered perovskites, consisting of alternating sheets of organic cations and inorganic metal halide layers, are a newly discovered family of 2D materials with a unique promise for energy and optoelectronic applications due to their strong light absorption and long photoexcited carrier lifetimes.^1−9^ In these systems, the confinement of charges in the inorganic quantum wells underpins a range of physical properties, making them ideally suited for room-temperature nonlinear operations in low-input power polaritonic devices and strong optical nonlinearities.^10^ Their potential role in optoelectronic applications is underpinned by a large photoluminescence (PL) quantum yield,^11^ tunable optical and electrical properties by composition,^12−16^ and narrow excitonic transitions with strong binding energies,^17^ to name a few. However, the inherent instability of these materials to a wide range of solvents has hindered their use in micro- and nanoscale devices. This scenario is now changing owing to the recent progress on the fluorination of the organic spacing layer, which has shown significant improvement of the 2D perovskites’ stability.^18−21^ These advances make 2D perovskites ripe for exploring real-life applications beyond the confines of energy harvesting.

In this article, we demonstrate the suitability of 2D-fluorinated phenylethylammonium lead iodide perovskite(F-PEA)_2_PbI_4_ (F-PEAI) for sensing in aqueous solutions and showcase their versatility in two real-life scenarios of key societal importance such as environmental monitoring and healthcare. The enhanced stability of these 2D perovskites supports the fabrication of photodetectors in ambient conditions using high-quality devices with outstanding figures of merit, such as a responsivity of >2200 A/W and a detectivity of 2.4 × 10^18^ Jones. At the same time, a unique encapsulation based on natural beeswax endows the F-PEAI sensors with an unprecedented resiliency to prolonged immersion in contaminated waters and a lifespan longer than one year. The biocompatibility of the encapsulant and its resilience make it possible to use this same device for monitoring the human heartbeat rate after rinsing off the contaminated waters simply with tap water, producing photoplethysmographs with clear systolic and diastolic signatures of the heartbeat.^22^ The unprecedented combination of the ambient stability and unique optoelectronic properties of 2D F-PEAI combined with the beeswax encapsulation paves the way to a new realm of opportunities for unexplored multipurpose applications key to sustainable electronics.

## Results and Discussion

**Figure 1:**
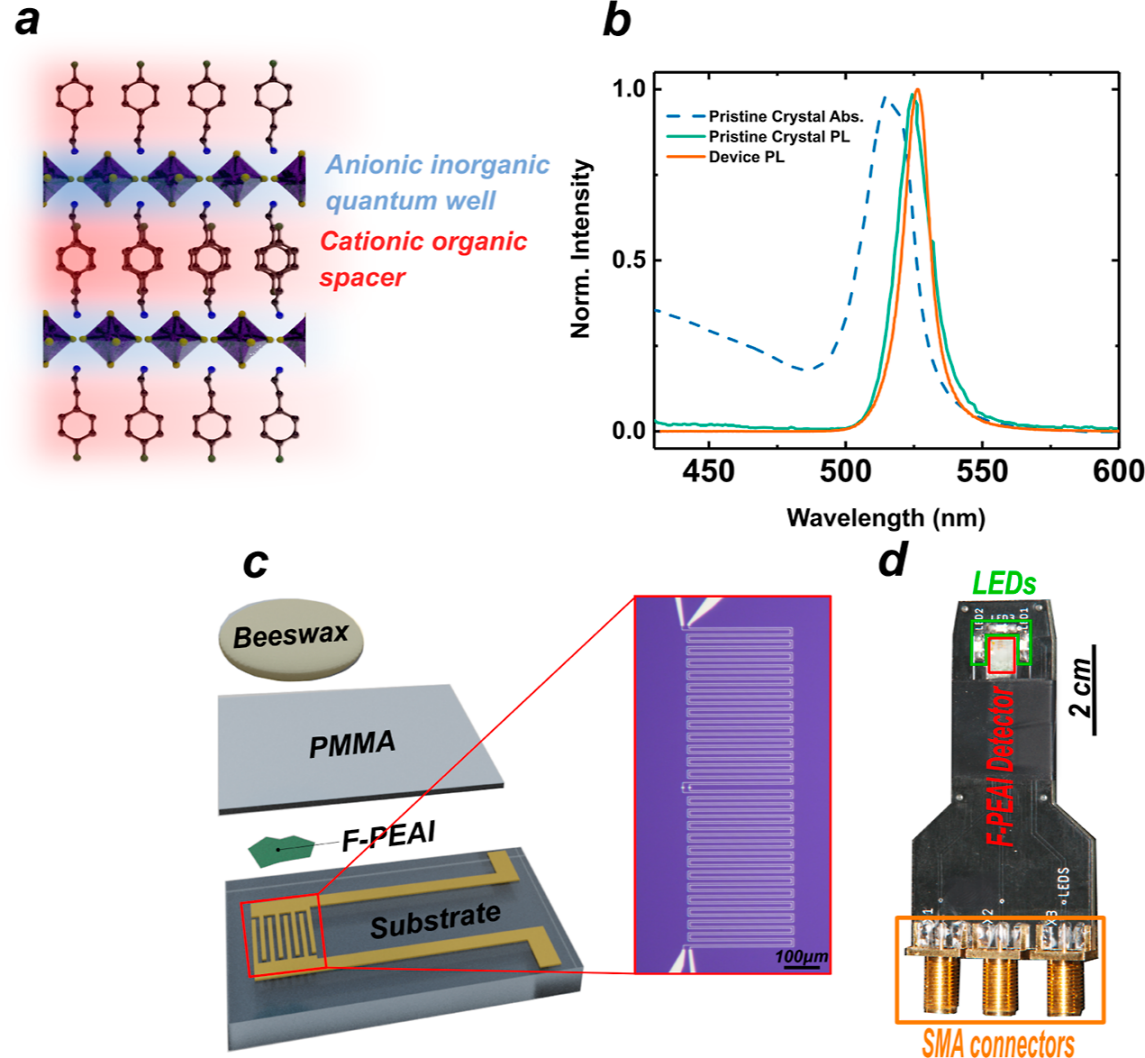
F-PEAI crystal composition, optical characteristics, and device structure. (a) Schematic of the layered 2D F-PEAI crystal structure. (b) Plot of the measured optical absorption (Abs.) in pristine F-PEAI and PL in a F-PEAI flake before and after encapsulation with PMMA/beeswax. (c) Exploded schematic of the encapsulated 2D F-PEAI-based sensor and a micrograph image of the serpentine metal contacts, see main text. (d) Encapsulated F-PEAI sensor mounted on a custom-made printed circuit board for measurements outside the laboratory environment, see Materials and Methods.

F-PEAI crystals were grown by the antisolvent vapor-assisted crystallization method,^23^ and their single crystalline phase was demonstrated in recent synchrotron X-ray diffraction studies.^24^ The crystal structure of this layered system consists of alternating sheets of inorganic  anions and organic alkylammonium, see Figure 1a. The inorganic layer governs the low-energy electronic excitations, while the organic spacer acts as a potential barrier leading to the confinement of charges in the plane of the inorganic layer, i.e., the quantum well.^14,16,25,26^ In F-PEAI, this confinement contributes to an energy bandgap of 2.61 eV (∼475 nm) and a large optical absorption in the blue to ultraviolet, while exciton physics dominates the absorption and PL spectrum at subgap energy up to room temperature with a peak at (∼523 nm), see Figure 1b. Earlier work has demonstrated that upon exposure to ambient moisture or solvents commonly used in the fabrication of semiconductor devices, the polar nature of perovskites drives the fast decomposition of the anionic metal-halide and cationic organic, making it difficult to fabricate high-quality devices in ambient conditions.^20,27^ Indeed, most of the demonstrated perovskite-based devices studied so far have relied on the processing in a moisture-free, controlled environment, such as that provided by a glovebox. The careful selection of hydrophobic cations in 2D perovskites can make these systems more resilient to ambient conditions.^28^ To this end, benzene-based cations result in a more organized and stable structure than those obtained with aliphatic species. Similarly, a different choice of the organic spacer molecule either by hydrogen bonding between organic layers or fluorination of the common spacer molecule C_6_H_5_C_2_H_4_NH_3_ (phenethylammonium, PEA) results in improved stability of these materials.^21,29−31^ The fluorinated spacer was also shown to influence the arrangement of the aromatic ring of PEA molecules in the 2D hybrid perovskite crystal, resulting in a better alignment of the inorganic layers and enhanced out-of-plane charge transport.^18^

Here, we fabricate in ambient conditions photodetectors based on F-PEAI following a process of mechanical exfoliation of thin crystals of ≤50 nm and their lamination onto quartz substrates with prepatterned interdigited 30 nm thick Au electrodes separated by 3 μm distance,^24^ see Figure 1c. The optical transparency of the quartz enables us to conduct accurate spectroscopic and optoelectronic characterization by shining light through the substrate. The ambitious goal of operating these devices in real-life wearable healthcare and environmental applications requires the encapsulation of with a biocompatible material that can withstand the harsh conditions of direct contact with bodily fluids and ambient contaminated waters. Inspired by the moisture barrier properties of beeswax and its wide use in the cosmetic, food, and pharmaceutical industries,^32^ we employ this natural material as a protective water barrier for perovskite photodetectors (see Supporting Information S1–S4). Figure 1b shows a measurement of the PL profile before and after the full assembly of the photodetector device (Figure 1c). It is apparent that the two spectra show no appreciable changes, confirming that the encapsulation process is not causing lattice distortion,^13,33,34^ changes to the crystal structure,^35,36^ or changes to the dielectric environment in the vicinity of the photoactive layers.^37,38^ A custom-built printed circuit board embedding lines with a capacitance of 160 pF (response time ≈10 ns) and integrated green light-emitting diode (LED) sources enables the multifunctional use of the device outside the lab environment, see Figure 1d.

**Figure 2:**
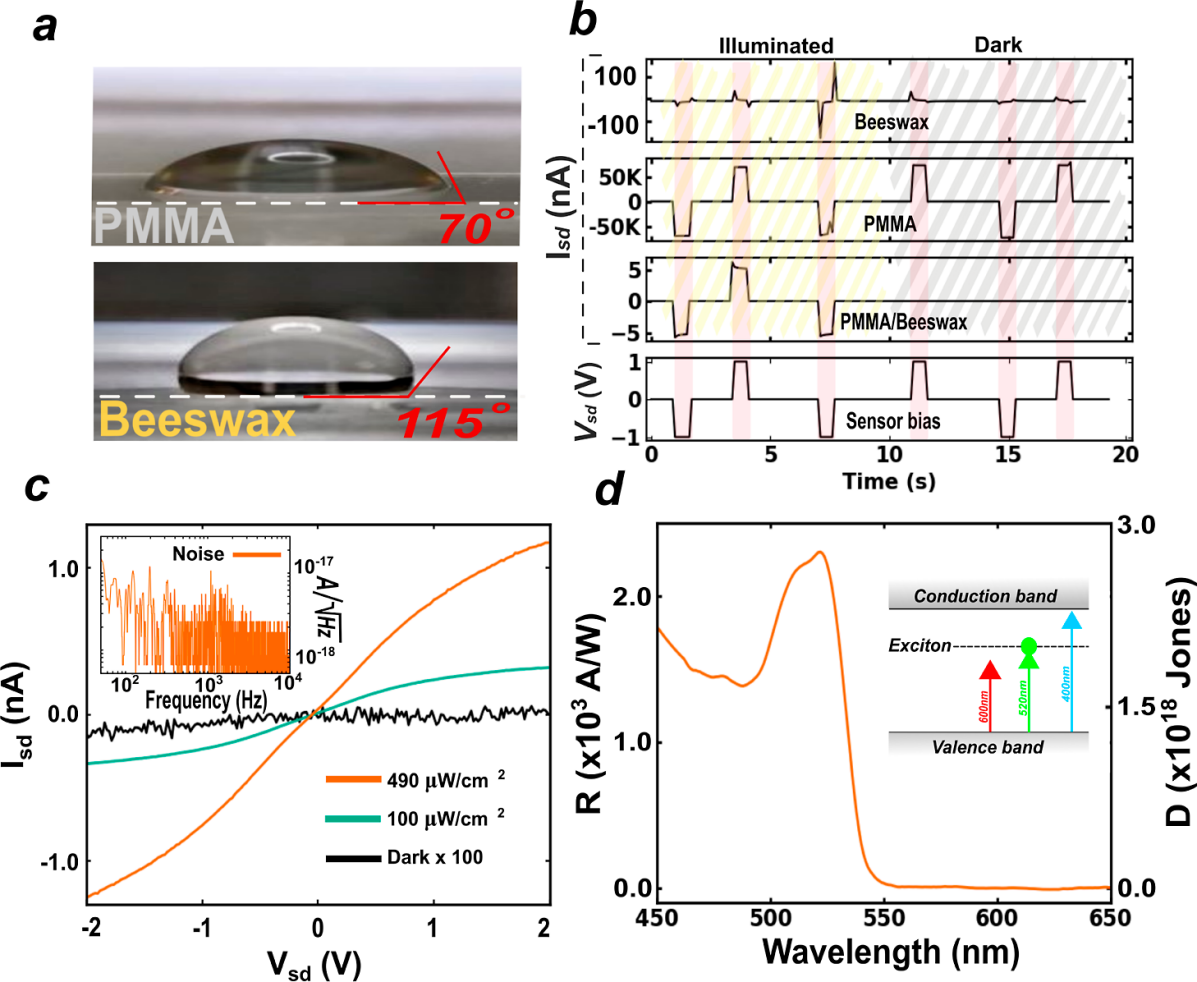
Contact angle measurements, beeswax and PMMA control, IV characteristics, noise spectral density, and spectral responsivity. (a) Contact angle measurements with a 10 μL microdroplet of deionized water on the surface of PMMA and beeswax. (b) Top three graphs show measured source drain current values under illumination (Thorlabs LED7WE, 15 mW) and dark for alternating pulsed *V*_SD_ applied to 2D F-PEAI photodetectors (bottom graph) submerged in deionized water and for three different types of encapsulations consisting of beeswax, PMMA, and PMMA/beeswax. (c) Plot of the source-drain current vs source-drain voltage bias for a representative 2D F-PEAI photodetector encapsulated by PMMA/beeswax in dark conditions and for different irradiances of a continuous wave laser (wavelength 514 nm and diameter 150 μm) and photoactive area 1.63 × 10^–5^ cm^2^. The inset shows the sensor noise spectral density measured at *V*_sd_ = 1 V illuminating the sample with the same laser with irradiance 490 μW/cm^2^. (d) Plot of the photoresponsivity vs wavelength for the F-PEAI sensor at fixed *V*_sd_ = 3 *V*. The photoactive area is 3.2 × 10^–4^ cm^2^, and this is illuminated by a monochromatic light beam of 0.27 cm^2^ and irradiance of ∼3 pW/cm^2^, see Materials and Methods and Supporting Information. The inset shows a diagram of the band edges of 2D F-PEAI and the exciton energy level.

Figure 2a shows contact angle measurements of deionized water on encapsulated devices in beeswax and polymethyl methacrylate (PMMA), i.e. a widely used polymer in electronics. We measure values of 115° for beeswax and 70° for PMMA, respectively, confirming the highly hydrophobic nature of beeswax^39^ as opposed to the hydrophilic PMMA.^40,41^ To assess whether the encapsulation in beeswax truly offers significant enhanced protection compared to that in PMMA, we conducted a comparative study of the resilience to water of 2D F-PEAI photodetectors encapsulated in (1) PMMA, (2) beeswax, and (3) PMMA/beeswax. Figure 2b shows the source-drain current (*I*_SD_) measured in response to an alternating pulsed source-drain bias (*V*_SD_) for each type of encapsulation while submerging the devices in water and under dark and illuminated conditions, with light shining through the quartz substrate to avoid light absorption by the translucent coating. An alternating pulsed *V*_SD_ is used to eliminate any spurious signal due to electrical conduction in the water. At the same time, the use of a pulsed *V*_SD_ is also ideally suited to reduce the device energy consumption in stand-alone field operations. Only the devices encapsulated in PMMA/beeswax show a photocurrent in response to pulses of the bias and a very low value of dark current, as expected for a semiconductor-based photodetector. On the other hand, the *I*_SD_ measured in the PMMA- and beeswax-encapsulated devices appears to be independent of the light conditions, suggesting an ionic origin which prevents the further use of the devices as sensors (see further details in Supporting Information S3). Figure 2c shows the bias-dependence of source-drain current measured in the dark and under illumination with a 514 nm continuous wave laser for two representative values of irradiance of 490 and 100 μW/cm^2^ and a photoactive area of 1.63 × 10^–5^ cm^2^. These measurements have been acquired using a home-developed low-noise integrated optoelectronic spectroscopy setup^42^ (see Materials and Methods) that is able to resolve the low level of dark current of the device at ∼1 pA. The presence of a Schottky barrier for electrons at the F-PEAI/metal contact underpins the nonlinear *I*–*V* characteristic at low bias. Upon increasing the bias, a semisaturation regime is reached when all the photogenerated carriers are extracted without recombining. The large photocurrent measured upon illuminating the device results in a large on/off ratio of >500, even at these modest values of irradiance. The device shows a low intrinsic electrical noise of ≃10^–17^*A*/ (see inset of Figure 2c), providing further evidence that the process of encapsulation does not adversely affect the 2D F-PEAI sensor. Finally, we characterize the spectral photoresponsivity of the photodetector using low-irradiance (∼3 pW/cm^2^) monochromatic incoherent light in the wavelength range from 450 nm up to 650 nm, see Figure 2c and Materials and Methods. At low irradiance, the photocurrent signal generated by the 2D semiconductor is not expected to suffer from light-induced charge trap saturation and can further benefit from internal gain due to charge recirculation boosting the photoresponsivity.^24,43,44^ This measurement reveals values as large as 2300 A/W when shining light of wavelength matching the exciton energy, and it generally exceeds 1500 A/W for photon energies larger than the single particle energy gap with a photodetectivity of 2.4 × 10^18^ Jones, see Figure 2d. Crucially, similar values of photoresponsivity and detectivity are measured in devices without encapsulation, confirming that the step of the encapsulation in PMMA/beeswax has no negative impact on the crystals consistently with the PL studies shown in Figure 1b, see Supporting Information S3 and S5.

**Figure 3:**
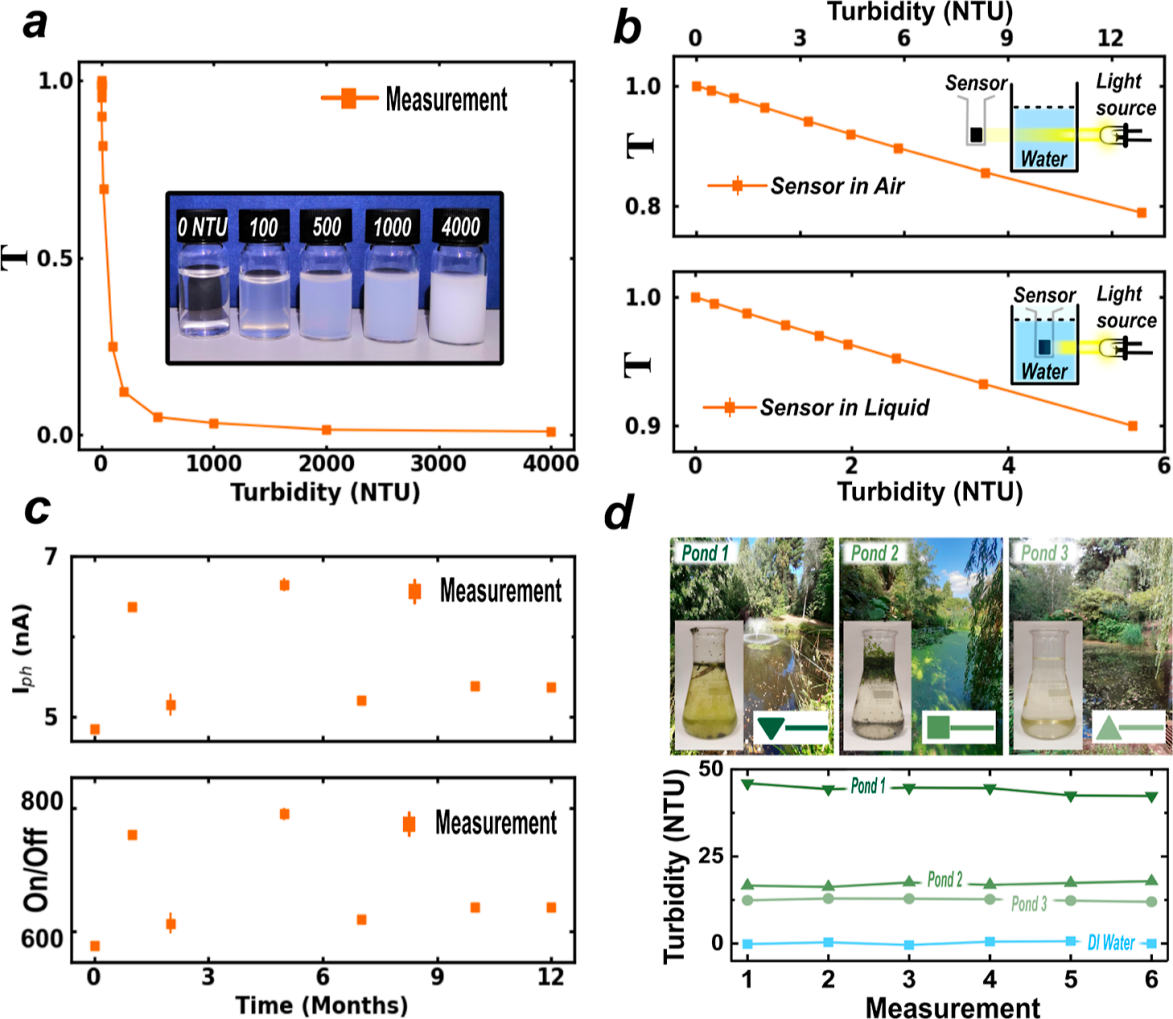
(a) Plot of the optical transmission of white light LED (Thorlabs LED7WE, 15 mW) through turbidity-calibrated standard (TURB4000, Merck Life Sciences) water samples measured with the PMMA/beeswax-encapsulated 2D F-PEAI. (b) Plots of the turbidity measurements of solutions of B6756 green ink (see Supporting Information S7) with the PMMA/beeswax-encapsulated 2D F-PEAI sensor in air and submerged in the B6756 solution in the range <12NTU. The insets show the setup used for each experiment. (c) Plot of the photocurrent, On/Off ratio, and % change in responsivity of the sensor acquired with the sensor submerged in water over a period of 12 months. The F-PEAI sensor was biased at *V*_sd_ = ± 1 *V*, and the white light LED was a 15 mW Thorlabs LED7WE. (d) Plot of turbidity measurements conducted using the beeswax-encapsulated 2D F-PEAI on 3 ponds in the Stretham campus of the University of Exeter and a comparative reading obtained measuring deionized water.

We now proceed to test the suitability of these detectors for water turbidity, i.e., a measure of transmitted light through a water sample, which is a widely used field monitoring technique for detecting the anomalous proliferation of cyanobacteria or blue-green algae which render water toxic to animals and humans.^45^ To this end, we conduct a first control experiment following the water turbidity industrial standards with a range of water solutions with Formazene Turbidity Standards (TURB4000, Sigma-Aldrich) attaining different clarity over the range of 0–4000 nephelometric turbidity units (NTU), see inset in Figure 3a. In these measurements, white light emitted (*V*_LED_ = 5 V) propagates through the turbid liquid, and it finally reaches the F-PEAI photodetector, generating a photocurrent (*I*_NTU_^ph^). The optical transmission (*T*) is given by *T* = *I*_NTU_^ph^/*I*_clear_^ph^, where *I*_clear_^ph^ is the photocurrent measured for the reference clear liquid. The large photoresponsivity and low electrical noise of these devices make the 2D F-PEAI sensors highly sensitive to a wide range of turbidities with a resolution of 0.075 NTU, rivaling the performance of high-end commercial turbidity sensors (see Supporting Information S5 and S6).

The stability, reproducibility, and overall suitability of the beeswax-encapsulated turbidity sensors are further tested by simulating analogous conditions to those of lightly contaminated water with small quantities of blue-green algae with <12 NTU. This is achieved by employing water solutions of commercial B6756 Merk green ink known to have a similar wavelength absorption to that of the algal growth (see Supporting Information S7). Figure 3b shows the measured turbidity for the same B6756/water solutions with the sensors submerged in the turbid liquid and in air. A similar linear scaling of the transmitted light is measured for the two conditions, demonstrating that the PMMA/beeswax encapsulation fully preserves the 2D F-PEAI photodetector. Figure 3c shows the values of photocurrent and on/off ratio of the device over a period of 12 months with a median photocurrent of 5.4 nA, with an overall change between the initial and final values of <7% (photocurrent) and <10% (on/off ratio), respectively. While testing the resilience in laboratory conditions is an important step for scientific progress, a true innovation breakthrough requires the testing of the technology in real-life conditions. To this end, we have tested the encapsulated calibrated 2D F-PEAI turbidity sensor in natural settings and conducted turbidity measurements of three different ponds on the campus of the University of Exeter, see Figure 3d scenarios, i.e. outside the laboratory environment. The stability of the sensor output for each of the ponds is evident when considering the small variation of six sequential readings with each measurement spaced 1 min apart.

**Figure 4:**
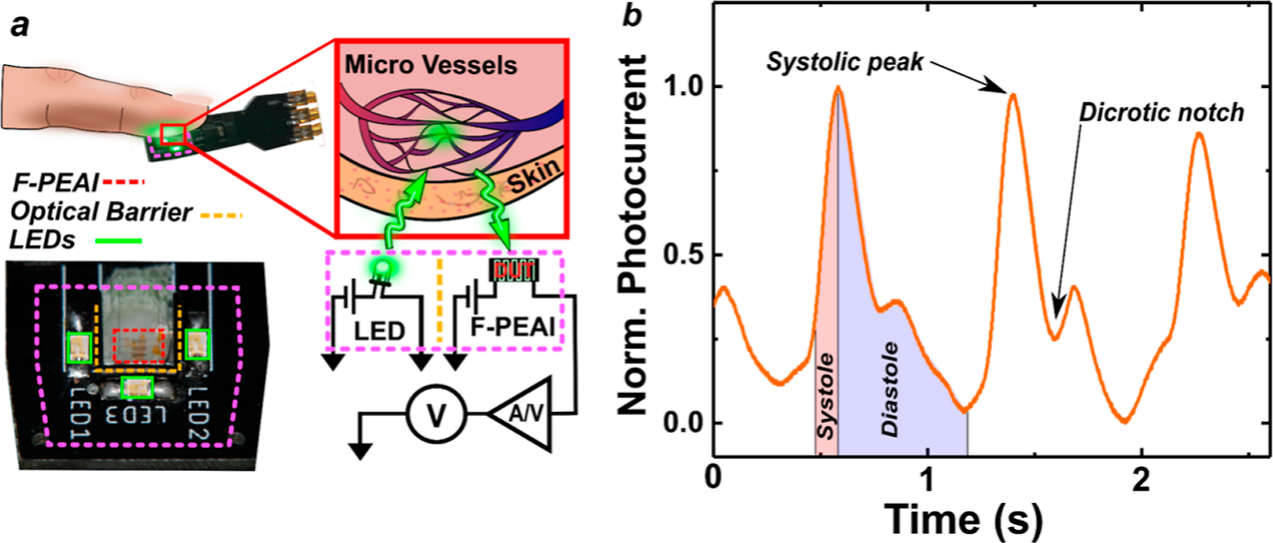
(a) Diagram of finger touching the integrated F-PEAI board for the acquisition of the PPG signal. Three green micro-LED lights emit light which interacts with the microvessels in the fingertip, gaining information on the heartbeat (right), which appears as a modulation of the reflected light intensity detected by the beeswax-encapsulated F-PEAI sensor, see Materials and Methods. (b) Normalized photocurrent measurement of heart rate detected using the F-PEAI-based sensor showing the systolic peak and dicrotic notch.

To test the extreme versatility of this encapsulation and explore its potential for enabling a true multipurpose range of applications for 2D F-PEAI sensors, we explore the possibility of using the same beeswax-encapsulated F-PEAI turbidity sensor for a widely used healthcare application such as photoplethysmography (PPG). This is widely used to provide information on a range of cardiac parameters through a simple measurement of changes to the absorbed or reflected light by the microvascular beds at peripheral body sites such as a finger.^46^ Owing to the integration and miniaturization of optoelectronic devices, PPG has become widespread in wearable electronics (e.g., smart watches and fit bands) with recent demonstrators based on graphene/quantum dots^47^ and 3D perovskites.^15^ Since the maximum pulsatile signal of the reflected light by the human body is in the range of 510–590 nm, the energy gap and spectral photoresponse of F-PEAI are ideally suited for the detection of PPG.^48^ To this end, we utilize a green LED light source (525 nm) commonly used in commercial fit bands and detect the back scattered light by a human finger, see Figure 4a and Materials and Methods. Hence, after washing away the water pollutants from the circuit board and F-PEAI turbidity photosensor with a simple rinse under tap water, the detector is placed in contact with a human finger. Figure 4b shows a time-resolved signal output of the 2D F-PEAI sensor biased with *V*_sd_ = 2 *V* with a 1 MΩ termination. The systolic and diastolic peaks are clearly distinguishable in the signal without any postprocessing, and in addition, our sensors clearly show other well-known critical points which can provide insights into different physiological trends such as arterial stiffness and help identify hypertension.^22^

## Conclusions

In conclusion, our experiments demonstrate the unexplored use of beeswax-encapsulated 2D-fluorinated phenylethylammonium lead iodide perovskite photodetectors for environmental and healthcare applications. The high crystal quality of F-PEAI and its fluorinated organic spacer make it possible to fabricate photodetectors with high figures of merit under ambient conditions. At the same time, the encapsulation by beeswax enables F-PEAI to withstand prolonged immersion in contaminated water and air for an extended period of at least 12 months with no measurable change in device performance. At the same time, the biocompatible nature of beeswax and its self-cleaning action make the very same F-PEAI photodetectors also compatible with epidermal direct contact, supporting the detection of heartbeat rate by means of PPG. Our results unveil the huge potential for the unexplored use of beeswax in optoelectronics, paving the way for sustainable electronics through the development of versatile and multipurpose devices supporting an unprecedented wide breadth of real-life applications with the same device and hand-held circuit solution. By reducing the need for multiple electronic gadgets, beeswax offers the opportunity to reduce electronic waste and secure a more sustainable future for electronics without compromising device performance.

## Materials and Methods

### Lithography of Prepatterned Contacts

Quartz substrates are coated with ∼400 nm of 950 K A6 PMMA and baked at 180 °C for 1 min. E-beam exposure is followed by sample development using a solution of IPA/MIBK in a ratio of 3:1 for 1 min 30 s and then rinsed in IPA. Metal deposition was achieved using e-beam evaporation of Ti/Au (5/30 nm). For metal lift-off, the sample was left in warm acetone (at 70 °C) for 1 h.

### (F-PEA)_2_PbI_4_ Single Crystals

F-PEAI single crystals were synthesized with the antisolvent vapor-assisted crystallization method carried out at room temperature. 267 mg of 4-fluoro-phenethylammonium iodide and 230.5 mg of PbI_2_ were dissolved in 1 mL of GBL and stirred at 70 °C for 30 min. A N_2_-filled glovebox was used to prepare the precursor solutions. Synthesis of 2D perovskite single crystals were achieved by the following steps: glass slides were cleaned with acetone and water in an ultrasonic bath for 10 min each before being heated to 80 °C for 10 min to remove organic contamination and finally rinsed 10 times in water. The perovskite solution (2 μL) was deposited on top of the substrate and immediately covered by the second glass substrate. A small vial containing 2 mL of dichloromethane (DCM) was next placed on top of the two sandwiched substrates. Next, the two sandwiched substrates and the vial containing DCM were placed in a bigger Teflon vial, closed with a screw cap, and left undisturbed for 12 h. After 12 h, millimeter-sized crystals of varying thicknesses (few to 10 μm) appeared between the two substrates.

### Encapsulation of F-PEAI Flakes in PMMA/Beeswax

PMMA (495 K A6 in anisole) is spin-coated to a thickness of 400 nm before baking at 60 °C for 20 min. Beeswax is applied by dip-coating the substrate with the flakes into molten beeswax (<80 °C) for 1 s before air-drying the substrate until the beeswax has set, following the recently established procedure demonstrated in beeswax-based triboelectric nanogenerators.^39^ See further details in Supporting Information S2.

### Optoelectronic Characterization

*Characterization with lasers.* The room-temperature spectroscopic, photocurrent, and real-time measurements were acquired using a custom-built optoelectronic characterization system optimized to probe the photophysical properties of 2D materials.^42^ The system embeds a number of solid-state laser sources (Coherent OBIS 375LX, 473LS, 514LX, and 561LS and Omicron LuxX 685, with powers ranging from 30 mW to 50 mW). Each laser is digitally modulated, and the power is adjusted using an analog signal. Custom-built drop-in-filter systems are used to introduce commercial neutral density, polarizers, notch, and bandpass filters in the optical path of the lasers and the microscope. The spectrometer is a Princeton Instruments Acton SP2500, equipped with three dispersion gratings (1200 g/mm with 500 and 750 nm blaze, and 1800 g/mm with 500 nm blaze), and it is equipped with a Princeton Instruments PIXIS400-eXcelon back-illuminated, Peltier-cooled, charge-coupled device camera. The optical path can be configured for Raman, PL, and transmission/reflection spectroscopy and laser light illumination for photocurrent maps simply by replacing or removing the appropriate filters. The sample stage is a Prior Scientific OptiScan ES111 instrument with a ProScan III controller with a minimum step size of 100 nm, enabling the accurate control of focused laser light for photocurrent maps. Calibrated power meters and fast photodetectors were used to measure the light intensity. *Characterization with monochromatic light.* Spectrally resolved photoresponse measurements were acquired using a xenon lamp and monochromator (Newport TLS300X) with light intensities adjusted using OD filters. All light source intensities were calibrated using a calibrated photodiode (Thorlabs S130CV). Electrical bias was achieved using a Xitron 2000 current and voltage source. Electrical signals were amplified using an Itaka 1300 current amplifier and captured using Agilent 34401a digital multimeters.

### Circuit Board

The encapsulated 2D F-PEAI sensor is mounted on a custom-developed circuit board. This contains three green micro-LED lights HSMM-C170—Broadcom with peak intensity at 525 nm and 1 mW of output optical power. Black optical barriers placed around the window of the 2D F-PEAI sensor stop the direct propagation of the emitted green light from the LEDs to the F-PEAI sensor.

### Turbidity Measurements

Turbidity measurements were carried out by placing the F-PEAI sensor in the light path of the white light LED (Thorlabs LED7WE *V*_bias_ = 5 V) with the light transmitting through the liquid to be measured. Either of two configurations were used depending on the experiment: the sensor submerged in the liquid (in the liquid container; Figure 3b bottom) or placed outside the liquid container (Figure 3a,c,d). The liquid container is a PET-based plastic container (400 mL). In the experiments, the container would be filled with 200 mL of the liquid to be measured. For the turbidity measurement, the sample would be biased by a timed alternating *V*_sd_ = ∓ 1 V using a Tektronix AFG1022 signal generator (see Figure 2b for the plot of *V*_sd_); *I*_sd_ was then amplified using a 1300 Itaka current amplifier before being sampled. Each data point was filtered using median filtering as it is computationally inexpensive and used in analog sensing applications.

### PPG Signal Measurement

PPG signal measurements were achieved by holding the F-PEAI sensor against the tip of the middle finger. The sensor was biased at *V*_sd_ = 2 V with the resulting signal amplified by a Femto DLPCA-200 transimpedance amplifier before being read by a Rohde & Schwarz 2 GHz Series 1000 digital oscilloscope.

## Ethical Approval

The authors declare that approval by the Research Ethics and Governance Department of the University of Exeter for the assigned study “Heartbeat rate bracelet fitness band” was obtained.

## Supporting Information Available

Perovskite crystal exfoliation and transfer techniques, additional data and description of the process of encapsulation, additional data on resilience tests of pristine and encapsulated F-PEAI crystal to liquids, detailed description of the contact-angle measurement setup, additional photodetector characterization, additional data for the turbidity sensing calibration, and additional data on the b6756 ink solution (PDF)
Correspondence should be addressed to S.R. (email: s.russo@exeter.ac.uk). All data needed to evaluate the conclusions in this paper are present in the main text and/or the Supporting Information. Additional data related to this paper are available from the corresponding authors upon reasonable request.
