# Supplementary material for: 2D Hybrid Perovskite Sensors for Environmental and Healthcare Monitoring
Source: ACS Appl Mater Interfaces. 2024 Jun 5;16(24):31399–406. doi: 10.1021/acsami.4c02966 (PMC11195008; doi:10.1021/acsami.4c02966)
Supplement: Supplementary file 1 — am4c02966_si_001.pdf [file am4c02966_si_001.pdf]

Supporting Information: 2D hybrid perovskite sensors for environmental and healthcare monitoring

Karl Jonas Riisnaes,[†] Mohammed Alshehri,[†] Ioannis Leontis,[†] Rosanna Mastria,[‡] Hoi Tung Lam,[†] Luisa De Marco,[‡] Annalisa Coriolano,[‡] Monica Felicia Craciun,[†] and Saverio Russo[*,†]

†*Centre for Graphene Science, College of Engineering, Mathematics and Physical Sciences, University of Exeter, Exeter EX4 4QL, United Kingdom*

‡*Institute of Nanotechnology, via Monteroni, 73100, Lecce, Italy*

E-mail: s.russo@exeter.ac.uk

S1: Perovskite crystal exfoliation and transfer techniques

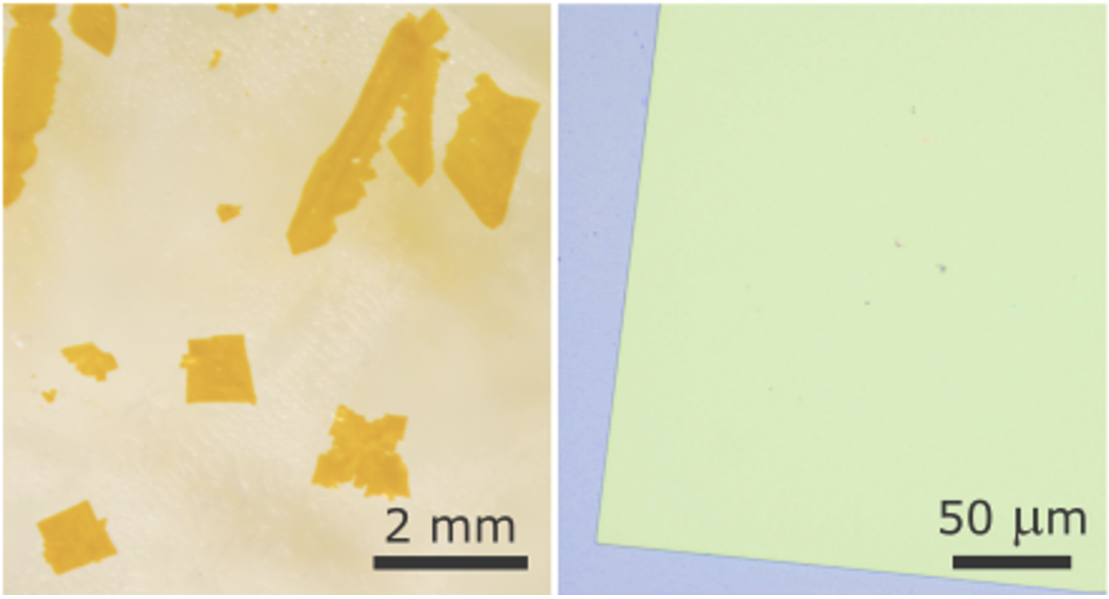

Fig. S1: Photograph (left) and optical micrograph (right) of 2D F-PEAI single-crystallyne flakes.

Figure S1 shows single crystals of 2D F-PEAI which are mechanically exfoliated using adhesive thermal-release tape (Graphene Supermarket, SKU: GTT-5P). Thin and uniform crystals are identified by inspection under a white light microscope (Nikon LV150). Hence, uniform, large and thin crystals which are attached to the tape are alligned onto a prepatterned substrate with gold contacts, and brought into contact with the substrate. Finally, the tape and substrate are heated to 95^oC for 10 seconds enabling the release of the substrate with the transferred flakes. The use of transparent quartz substrates facilitates the process of aligning the prepatterned contacts with suitable flakes, though transfer on opaque substrates is also possible by viewing the gold pads through the thermal release tape. Figure S2 shows the atomic force microscope topographic image of a representative 2D F-PEAI photodetector, and a height line cut across the 50 nm Au electrode and the 50 nm 2D F-PEAI crystal.

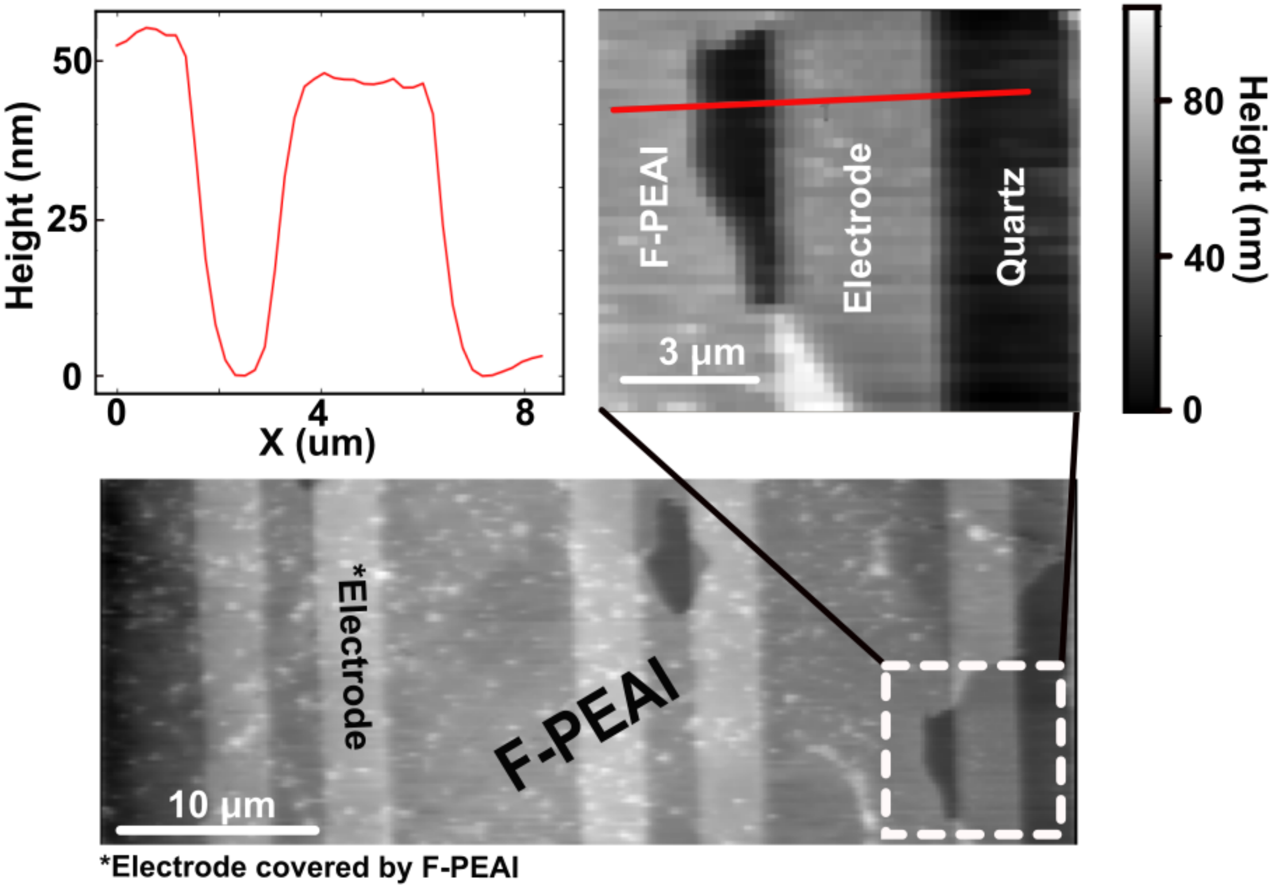

Fig. S2: Atomic force microscopy imaging of F-PEAI based photodetector with device profile line-scan. The cropped area line-scan clearly shows the F-PEAI crystal and electrode thickness.

S2: Process of encapsulation

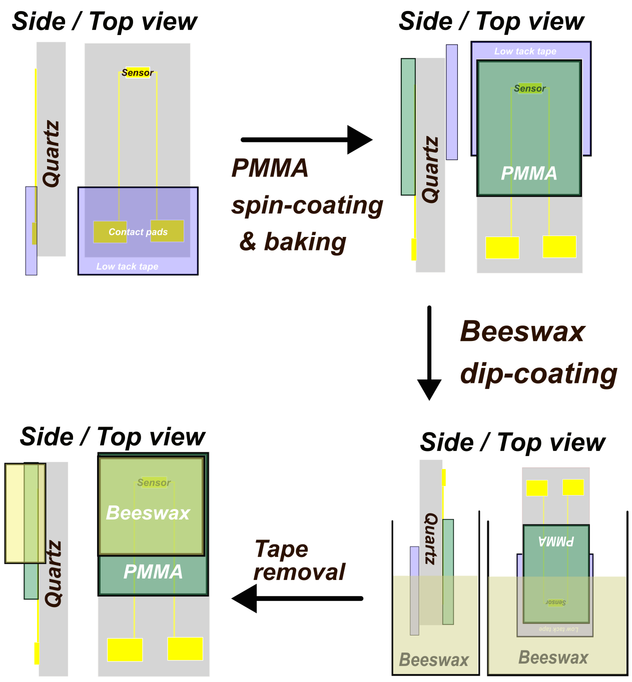

Fig. S3: Process of encapsulating F-PEAI sensors photodetectors in PMMA/beeswax

Figure S3 shows a schematic view of the key steps undertaken in the encapsulation of 2D F-PEAI photodetecotrs. At first, the electrodes contact pads are covered with low tack tape (Loadpoint, LP005442) to protect them from being covered by the electrically insulating coating layers such as Poly(methyl methacrylate) (PMMA) and beeswax. Hence, PMMA (495K A6 in anisole) is spin-coated and baked at 60°C for 20 minutes on a hotplate, attaining a thickness of 400 nm as measured by a profilometer (Dektak: Alphastep D100), see Fig. S4. The beeswax is applied by hot deep coating using a dip coater (Ossila) set at insertion and withdrawal speeds of 1 mms^{-1} and a dwell time of 2 s whilst the beeswax is melted at 80^oC, following a procedure optimised for beeswax triboelectric nanogenerators.[1] This results in an $\approx$ 200μm thick beeswax as measured by a profilometer (Dektak: Alphastep D100), see Fig. S4. The thickness of the beeswax is controlled by the withdrawal speed and by diluting

the beeswax with toluene as described in the work by Kovalska et al.[1] The use of a thick beeswax layer is preferable in order to reduce failure of the device due to the presence of throats and trenches occurring in the beeswax layer. In the dip coating process the bottom side of the substrate is protected by a tape and the curing of the beeswax is conducted in ambient conditions. In the final step, the masking tape of the contacts is removed.

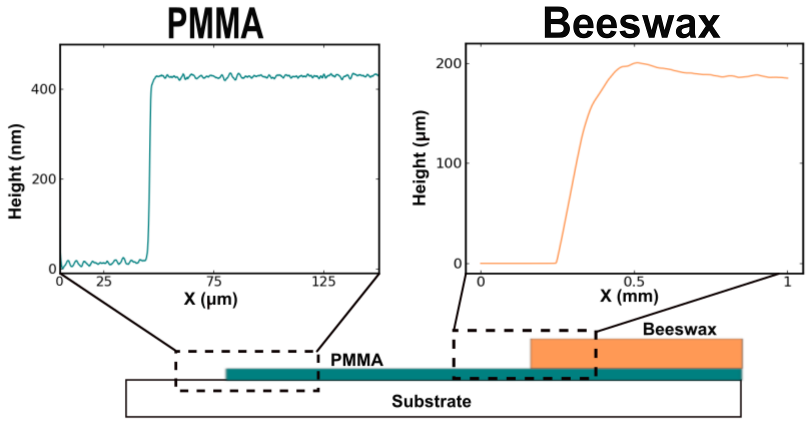

Fig. S4: Height profile measurements of PMMA and beeswax

S3: Additional resilience tests of pristine and encapsulated F-PEAI crystal to liquids

Tests of resilience to water of pristine and PMMA encapsulated F-PEAI are conducted by submerging the samples in de-ionised water at room temperature and monitoring their degradation with the direct visual inspection under an optical microscope, see Fig S5 1a-b. After 1 hour F-PEAI crystals were completely dissolved (see Fig. S5), whilst those covered by PMMA dissolved after 48 hours, see Fig. S5. Figure S6 shows photoluminesence (PL) measurements of the samples before (blue) and after (black) submerging the samples in de-ionised water and water solutions of Merk ink, as well as formazine for a total of 48 hours (Fig S6 a, c, e, g - uncovered crystals. Fig S6 b, d, f,h - crystals covered in PMMA) The

complete disappearence of any PL signal confirms that the crystals have dissolved.

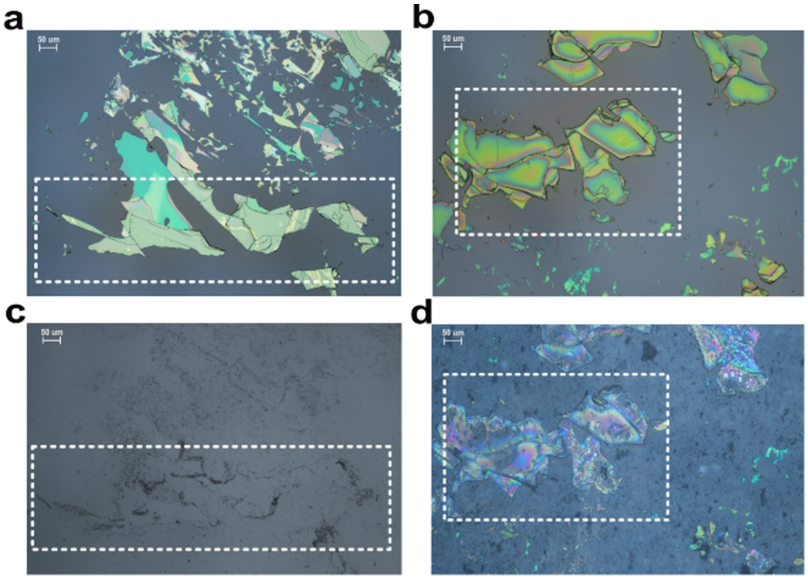

Fig. S5: (a) Micrographs of a pristine F-PEAI crystal. (b) Micrograph of a F-PEAI crystal coated by 400 nm of PMMA following the steps described in the main text. (c) Micrograph of the sample shown in panel (a) after dipping in water for 1 hour. (d) Micrograph of the sample shown in panel (b) after dipping in water for 48 hours. The dashed white boxes highlight regions of interest.

Figure S7 shows the photocurrent measured in 2D F-PEAI sensors with different encapsulations (i.e. PMMA, beeswax and PMMA/beeswax) in air and water. In all cases a constant illumination by an LED light (Thorlabs LED7WE) is used and the 2D F-PEAI is biased by an alternating $V_{sd} = \pm 1$ V. It is apparent that a clear photo response for submerged samples in DI water is only measured in sensors encapsulated by PMMA/beeswax and no measurable change in behaviour between operation in air and in water is observed, demonstrating the unique value of this encapsulation. Figure S8 shows the measured source-drain current in control experiments for the electrodes without any 2D F-PEAI. The electrical conductivity of water supports the flow of a large source-drain current in response to a source-drain bias applied to the electrodes in the absence of any encapsulation. When PMMA is applied onto the electrodes, water infiltrates the polymer (as seen by the full degradation of 2D F-PEAI coated by PMMA when immersed in water, see Figure S5 and S6) leading to a non zero source-drain current in response to an applied bias. The equivalent circuit for the system of

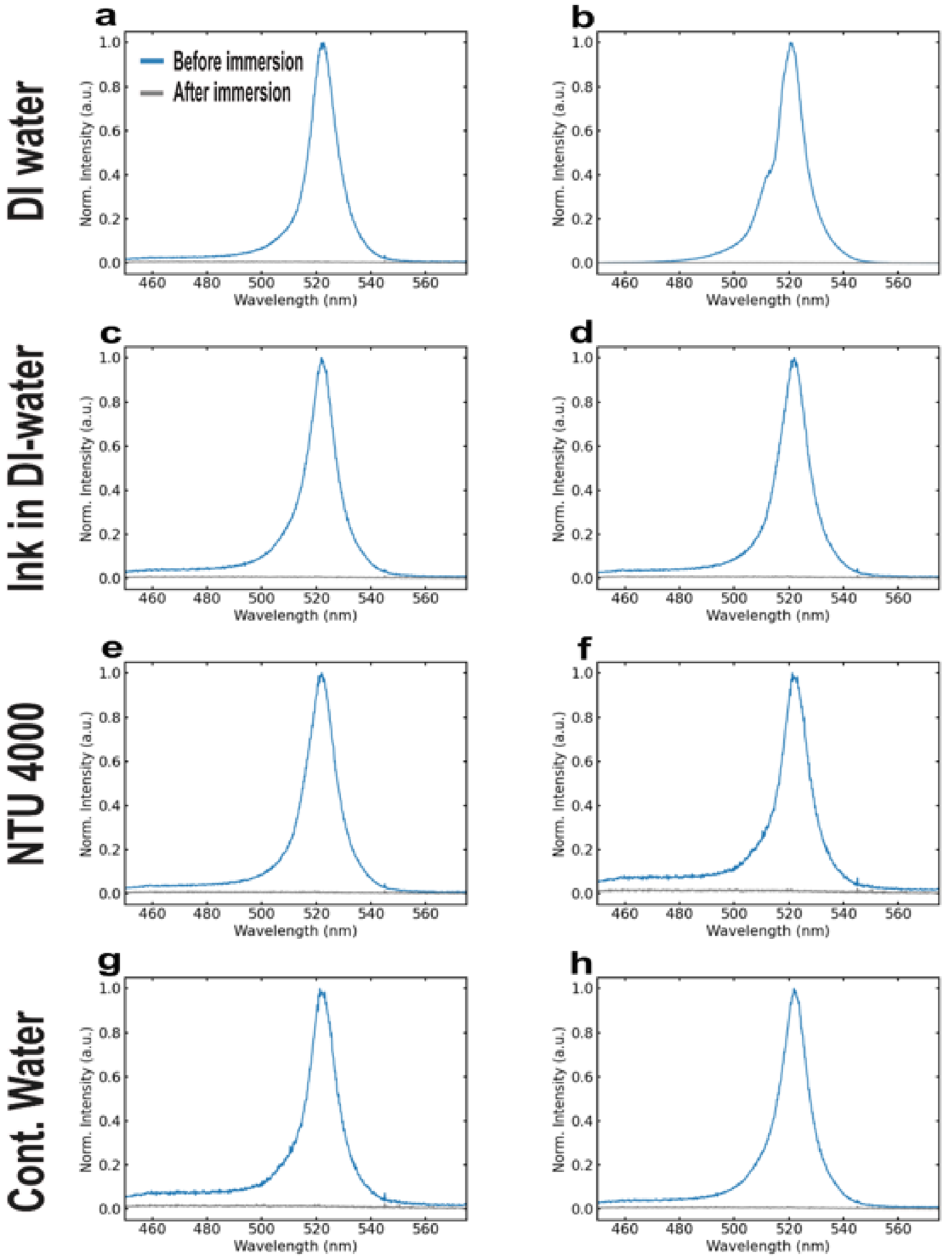

[H]

Fig. S6: (a-h) Photoluminescence spetra for pristine F-PEAI in panels (a, c, e, g) and F-PEAI coated by PMMA in panels (b, d, f, h) before (blue) and after (gray) dipping in de-ionised (DI) water (a,b), dispersed ink in DI-water (c,d), formazene (NTU4000) in DI water (e,f), and contaminated water natural water from pond number 3 (g,h). All samples are fully degraded after 48 hours of immersion.

electrodes encapsulated in a thin beeswax (<3μm) and submerged in water is the parallel of two resistors, i.e. one for the electrical path in the beeswax and a second resistor for the water with the latter being the lowest resistive channel, respectively. The presence of charge traps in the beeswax enables the hopping of charges from the metal electrodes to the water, which will then support the further flow of current to the drain electrode. For this reason, electrodes encapsulated solely by beeswax do not exhibit a significant source-drain current in air, where the dry conditions force the flow of charges only through the beeswax. Finally, to prevent current from flowing into the parallel conduction path of the water, we introduce the additional PMMA layer underneath the beeswax. In this case, the flow of current towards the water is fully suppressed owing to the dielectric properties of PMMA, whilst the hydrophobic nature of beeswax prevents water from infiltrating the PMMA, effectively preserving the dielectric properties of the PMMA and the semiconducting 2D F-PEAI.

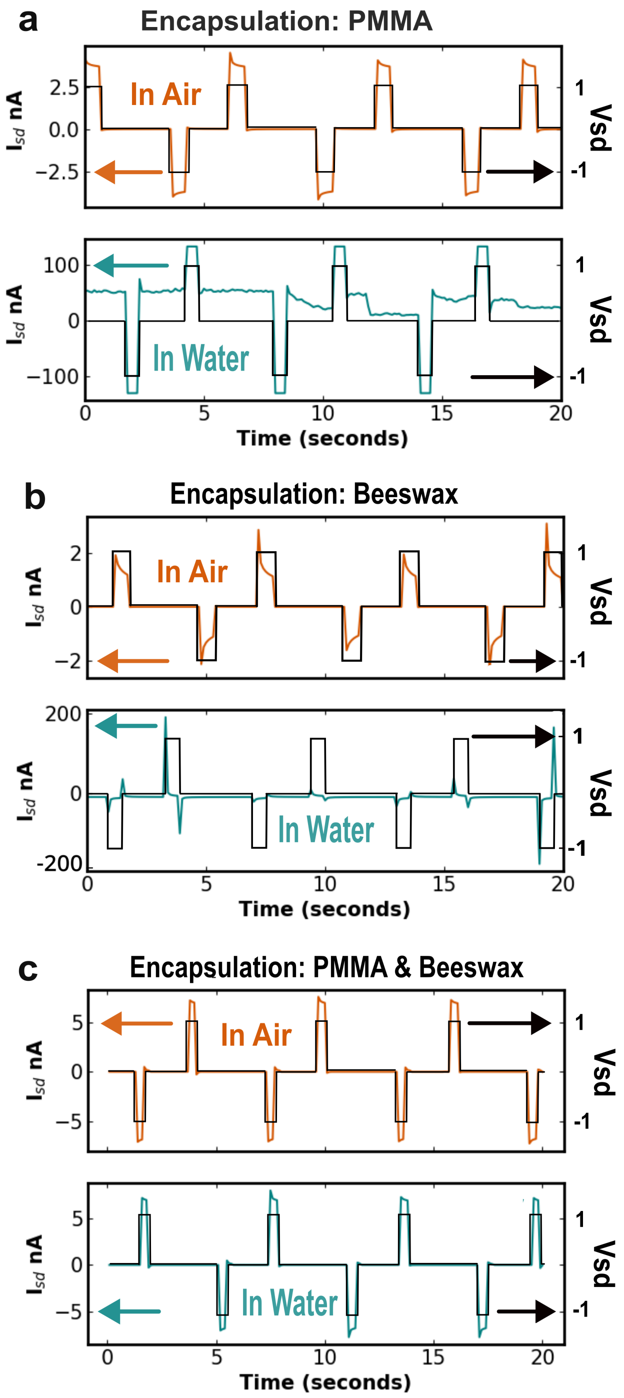

Fig. S7: Partially and fully encapsulated F-PEAI photodetectors in air and submerged in water. All detectors are under constant illumination (LED light (Thorlabs LED7WE)) and biased at ±1 V. (a) F-PEAI detector encapsulated in PMMA, (b) F-PEAI detector encapsulated in beeswax and (c) F-PEAI encapsulated in PMMA and beeswax.

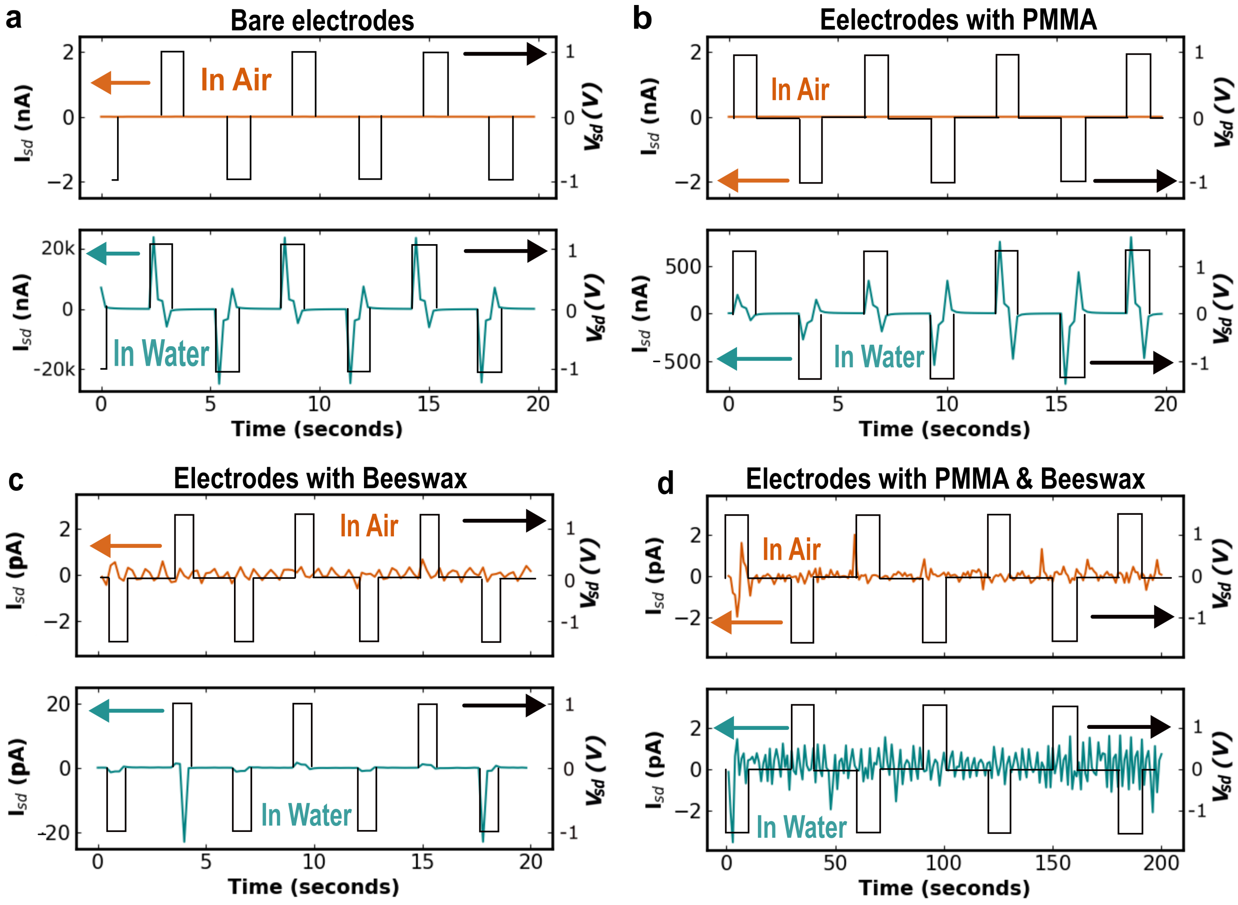

Fig. S8: Bare and covered (PMMA and beeswax) serpentine electrodes exposed to air and DI-water whilst under an alternating source-drain voltage (V_{sd}). (a) Bare electrodes. (b) Electrodes covered by PMMA (c). Electrodes covered with beeswax. (d) Electrodes covered with PMMA and beeswax

S4: Contact-angle measurement setup

The contact angle measurements were acquired using a commercial Ossila Contact Angle Goniometer equipped with a high resolution camera (see diagram Fig: S9). A 10 μl micro-droplet of de-ionised water was placed on the surface of beeswax and PMMA, and a grazing angle photography was acquired. Hence, the image captured of the droplet was then imported into a CAD software to calculate the angle between the surface and the droplet. The powerful, mains-powered monochromatic backlight helps to accurately detect the edges of the droplet. The bright, uniform rectangular panel creates a larger change in pixel lightness than would otherwise occur, which ensures that a sharp edge can be found. The fine level of adjustment of the sample stage enables the user to set a slight tilt towards the camera to help align the baseline, which we further check with a calibrated spirit level. In our experiment we can align the baseline to $<1°$(white dashed line in Fig. 2a) and the accuracy in the contact angle measurement is of $\pm 1°$, as also specified by the manufacturer.

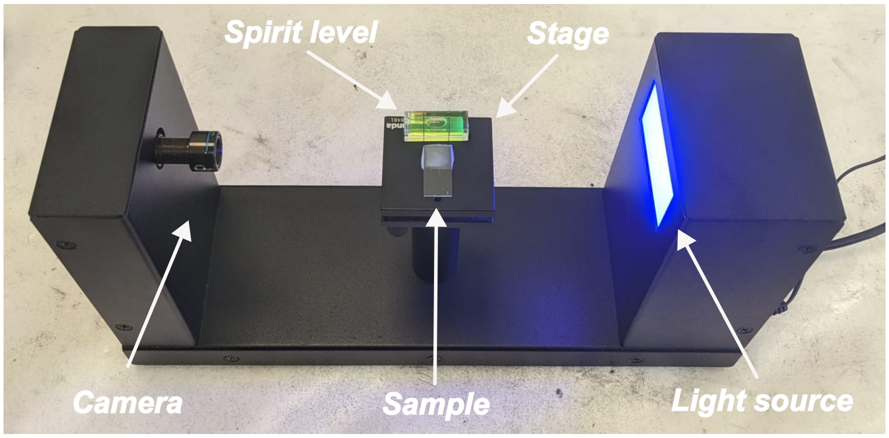

Fig. S9: Picture of the set-up for the contact angle measurements (Ossila: Contact Angle Goniometer).

S5. Photodetector characterization

To characterize the sensitivity of the 2D FPEAI photodetector we characterize the spectral responsivity (R). This is a figure of merit capturing the electrical output generated by the

sensor per incident optical input on the given photoactive area. The equation used for the responsivity is $R = J_{ph}/P_{light}$, where J_{ph} is the photocurrent density and P_{light} is the irradiance of the incident light. The external quantum efficiency (EQE) of the photodetector is a figure of merit which provides insight on the device efficiency in the generation and collection of charges upon photon absorption for a given source-drain bias. This is given by the expression $EQE = (Rh\nu)/e$ where R is the responsivity, $h\nu$ is the photon energy and e is the electron charge. Finally, the specific detectivity (D^*) is given by $D^* = (R\sqrt{Af})/S_n$ where R is the responsivity, A is the photo-active area, f the bandwidth, and S_n is the noise spectral density of the device. Table 1 shows a comparison of some of the key figures of merit for 2D F-PEAI against the state-of-the-art perovskite photodetectors. The photo-active area of the device is measured with high accuracy by rastering a focused continuous wave laser (spot size = $\sim 0.5\ \mu$m, of wavelength = 514nm and irradiance = 4.8W/cm^2) onto the sample. In our set-up[2] the laser beam is kept fixed on the principle optical axis, whilst the stage of the sample is moved with micro-motors in steps of 3μm. Figure S10 shows a representative photocurrent acquired at fixed source-drain bias of 2 V measured in the device presented in Figure 2 of the main manuscript. Evidently, only the photoactive area of the device gives a photoresponse upon illumination. Finally, Table S2 shows the figures of merit measured in five non-encapsulated F-PEAI based photodetectors at room temperature with the devices in vacuum. The performance of these photodetectors is similar to that of the encapsulated devices in PMMA/beeswax, providing strong evidence that the encapsulation does not adversely affect the devices. The measured time response for the encapsulated and non-encapsulated devices mounted on the circuit board of Fig. 1 is of approximately 10 ns. This time response is limited by the capacitance of the circuit board (measured to be ≈ 160 pF) rather than by the F-PEAI photodetectors (< 1 pF), leaving room for improving the time response with an optimised circuit design.

Perovskite	Device type	D* (Jones)	R (A/W)	On/Off Ratio	Ref
F-PEAI	SC	$2.4x10^{18}$	$2.3x10^{3}$	$6x10^{2}$	This study
$MAPbI_3$	SC-W	$1.7x10^{13}$	3.87	$4.3x10^{3}$	[3]
$BA_2(MA)_3Pb_4I_{13}$	SC-B	$1.2x10^{15}$	$7.4x10^{4}$	10^{5}	[4]
$(PEA)_2PbI_4$	SC-M	$1.6x10^{15}$	98.17	NR	[5]
$(PEA)_2PbI_4$	SC-FL	$1.2x10^{13}$	0.46	$1.6x10^{6}$	[6]
$BDAPbI_4$	SC	$1.2x10^{11}$	0.927	$2x10^{4}$	[7]
$(PEA)_2PbI_4$	SC	$1.9x10^{15}$	139.6	NR	[8]
$(PA)_2(G)Pb_2I_7$	SC-B	$6.3x10^{12}$	47	$2.5x10^{3}$	[9]
$(ThMA)_2(MA)_2Pb_3I_{10}$	SC-NW	$9.1x10^{15}$	$1.1x10^{4}$	NR	[10]
$MAPbI_3$	SC-FL	$1.5x10^{13}$	0.32	NR	[11]
$MAPbBr_3$	SC-FL	$6.6x10^{11}$	$5.6x10^{3}$	200	[12]
$MAPbI_3$:F8IC:PTB7-Th	TF	$2.3x10^{11}$	0.43	NR	[13]
$MAPbI_3$	TF	$1x10^{12}$	0.55	10^{4}	[14]
L-AA $FAPbI_3$	TF	$1x10^{12}$	0.45	NR	[15]
$CsPbBr_3$	TF	$1.6x10^{13}$	0.02	NR	[16]
$CsPbIBr_2$	TF	$9.7x10^{12}$	0.28	NR	[17]
$CsPbBr_3$	TF	$4.8x10^{12}$	0.172	$1.3x10^{5}$	[18]
$CsPb_xS_{n1-x}(Br_yI_{1˘y})_3$	NW	$2x10^{10}$	0.01	NR	[19]

Table S1: Summary table of the published perovskites photodetectors to date (MA=Methylammonium; BA=Butylammonium; PEA=Phenethylammonium; BDA=1,4-butanediammonium; PA=n-pentylaminium; G=guanidinium; ThMA= 2-thiophenemethylammonium; L-AA = L-ascorbic acid; FA=Formamidinium; SC=Single crystal; W=Wafer; B=Bulk; M=Membrane; FL=Few layer; NW=Nano wire; TF=Thin film)

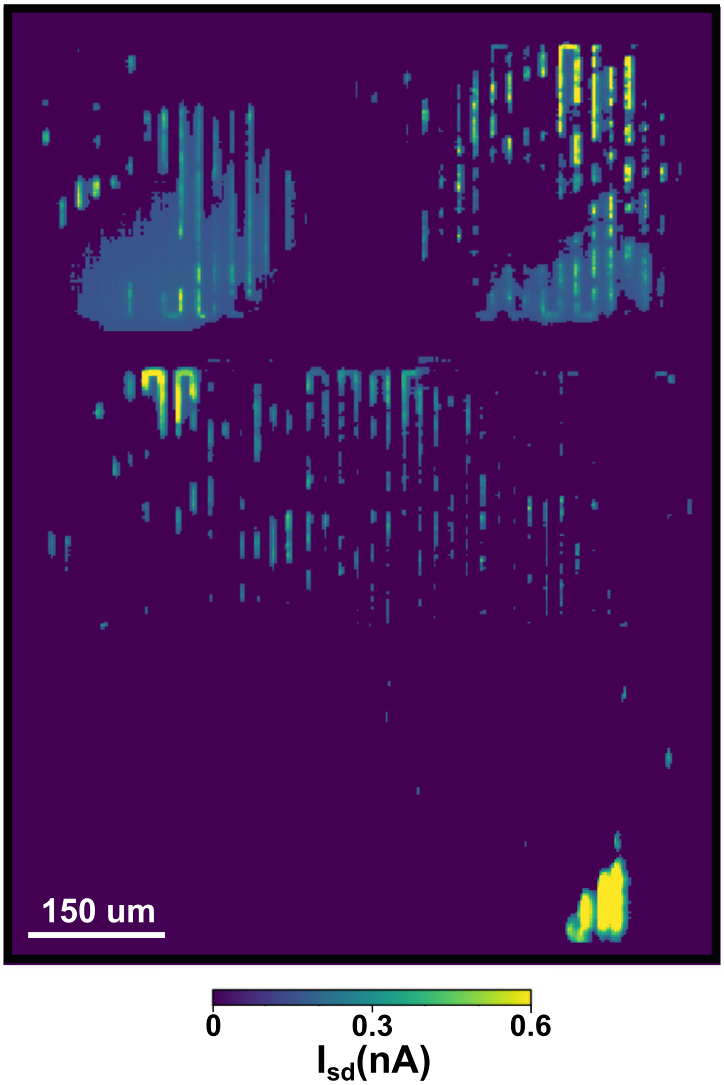

Fig. S10: Spatially resolved photocurrent map for the device shown in Figure 2 of the main manuscript. The laser spot size = ~ 0.5 μm, with a wavelength = 514nm and irradiance = 4.8W/cm^2). The sample was biased at V_{sd} = 2V. The estimate of the total active area is 3202x10$^-$4 cm^2 with a cutoff of contributing photocurrent set to 500 fA (i.e. noise-floor)).

Non-encapsulated F-PEAI photodetectors performance				
Sample	Responsivity (A/W)	Sn ($A/\sqrt{Hz}$)	D* (Jones)	Photoactive area (cm^2)
1	2169.7	5.3×10^{-17}	4.5×10^{17}	1.22×10^{-4}
2	2817.16	1.8×10^{-17}	1.22×10^{17}	6.4×10^{-5}
3	1442.38	2.3×10^{-17}	4.2×10^{17}	5×10^{-5}
4	1728.14	5.6×10^{-18}	2.1×10^{18}	4.9×10^{-5}
5	2754.4	2.7×10^{-17}	6.15×10^{17}	3.8×10^{-5}

Table S2: Summary of responsivity, noise spectral density and photodetectivity measured in five non-encapsulated F-PEAI based photodetectors. The values were measured with a 514 nm continuous wave laser with an irradiance of $\sim$ 5x10^{-12} W/cm^2. All devices were biased at V_{sd} = 5V

S6: Turbidity sensing calibration.

The calibration for the turbidity measurements was carried out by using Formazene Turbidity standard (TURB4000, Sigma Aldrich) in a transmittance configuration. In this experiment the tranmitted fraction of white LED light (Thorlabs LED7WE) propagating through the liquid is absorbed by the F-PEAI sensor generating a photocurrent (I^{ph}_{NTU}) . Figures S11 and S12 show plots of the optical transmission (T) for turbidity ranges up to 100 NTU and 2 NTU, respectively. The transmission is obtained from a direct measurement of the photocurrent using the relation $T = I^{ph}_{NTU}/I^{ph}_{clear}$ where I^{ph}_{clear} is the measured photocurrent for the reference clear liquid. The experimental data are described by the established exponential function [20] $I_0 = m\,e^{-nT} + T_0$ with fitting parameters $m = 0.81183 \pm 5\times10^{-6}$, $n = 1/42.9815 \pm 5\times10^{-8}$ and $T_0 = 0.18018 \pm 5\times10^{-6}$. This calibration is used to determine the turbidity of the water in the ponds shown in Figure 3d of the main manuscript. Finally, Table S3 shows a comparison of the F-PEAI based turbidity sensor precision against that of other competing technologies.

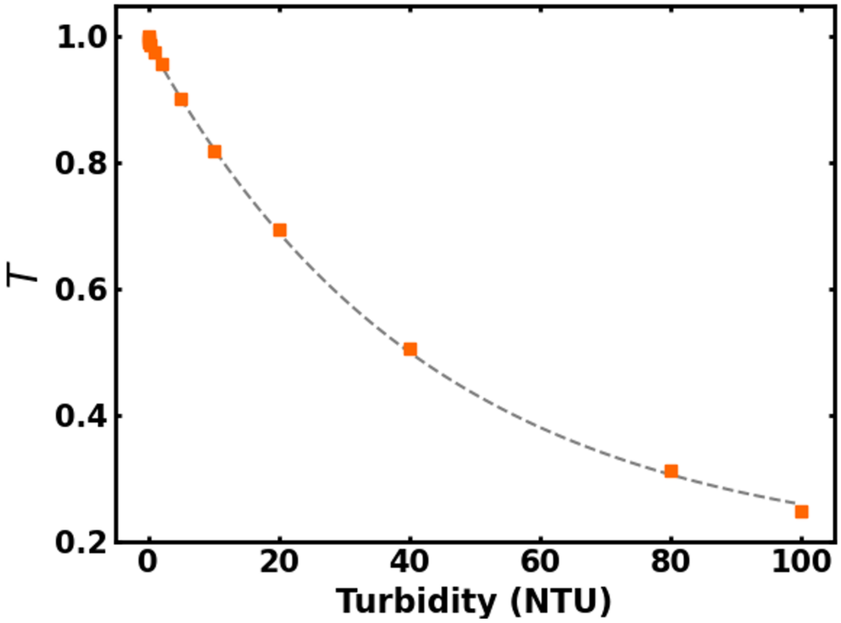

Fig. S11: Plot of the turbidity measurements (orange square) used to calibrate the sensor using a fit to the exponential function $me^{-nT} + T_0$ described in the text.

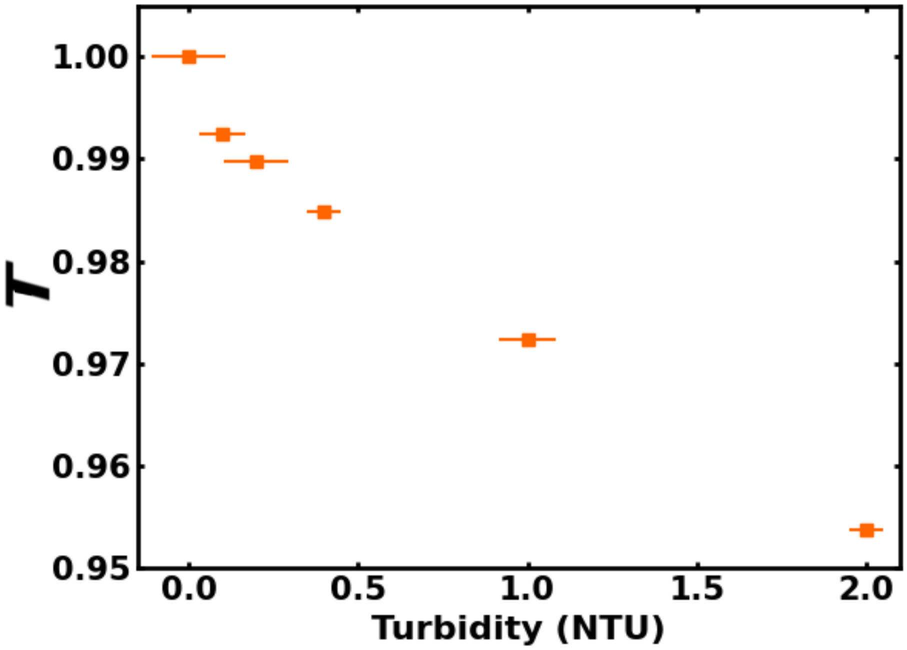

Fig. S12: Figure showing turbidity calibration data points for low turbidity solutions (in DI water).

Table S3: Table of commercial turbidity sensors.

Turbidity sensors		
Sensor Name	Precision (NTU)	Reference
F-PEAI based sensor	0.075	This study
INW Turbo Turbidity Sensor	2	[21]
Vernier Turbidity Sensor	0.25	[22]
Aqualabo Turbidity Sensor	0.01 - 1	[23]
Valeport Hyperion Turbidity Sensor	0.03	[24]
Real Tech Turbidity Sensor	1	[25]

S7: b6756 green ink solution

To test in a laboratory environment the suitability of the 2D F-PEAI photodetector for sensing the potential presence of cyanobacteria in water, we selected a commercial water soluble green ink (Brilliant green inc, b6756, Merck Life Sciences) with a similar visible wavelength absorption to that of common cyanobacteria, see Fig. S13. The ink granules were dissolved in de-ionised water with a ratio of 2 mg : 1 mg of water : b6756. After mixing using a glass rod the ink solution was heated to 60 degrees for a total of 2 hours to fully dissolve any granule.

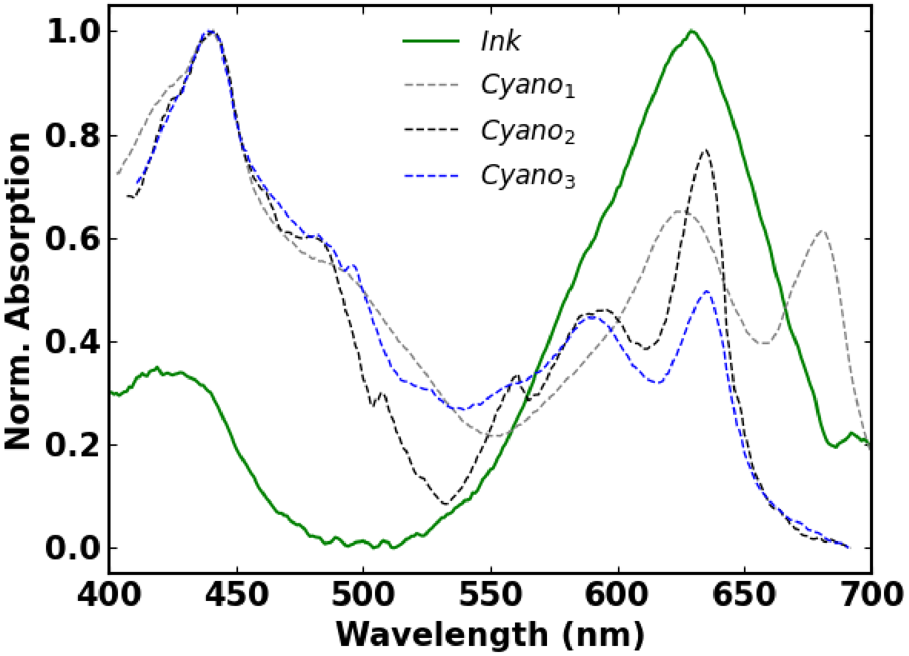

Fig. S13: Absorption spectrum of 2 mg : 1 mg of water : b6756 solution. For comparison the absorption spectra of 3 common cyanobacteria: Synechocystis ($Cyano_1$ - gray), Synechococcus sp ($Cyano_2$ - black) and Oscillatoria sp. ($Cyano_3$ - blue) are provided for reference.[26,27]

References

1. Kovalska, E.; Lam, H. T.; Saadi, Z.; Mastria, R.; Neves, A. I. S.; Russo, S.; Craciun, M. F. Textile beeswax triboelectric nanogenerator as self-powered sound detectors and mechano-acoustic energy harvesters. *Nano Energy* **2024**, *120*, 109109–109109.

2. Sanctis, A. D.; Jones, G. F.; Townsend, N. J.; Craciun, M. F.; Russo, S. An integrated and multi-purpose microscope for the characterization of atomically thin optoelectronic devices. *Review of Scientific Instruments* **2017**, *88*, 055102.

3. Gao, J.; Liang, Q.; Li, G.; Ji, T.; Liu, Y.; Fan, M.; Hao, Y.; Liu, S. F.; Wu, Y.; Cui, Y. Single-crystalline lead halide perovskite wafers for high performance photodetectors. *Journal of Materials Chemistry C* **2019**, *7*, 8357–8363.

4. Leng, K.; Abdelwahab, I.; Verzhbitskiy, I.; Telychko, M.; Chu, L.; Fu, W.; Chi, X.; Guo, N.; Chen, Z.; Chen, Z.; Zhang, C.; Xu, Q. H.; Lu, J.; Chhowalla, M.; Eda, G.; Loh, K. P. Molecularly thin two-dimensional hybrid perovskites with tunable optoelectronic properties due to reversible surface relaxation. *Nature Materials* **2018**, *17*, 908–914.

5. Liu, Y.; Zhang, Y.; Yang, Z.; Ye, H.; Feng, J.; Xu, Z.; Zhang, X.; Munir, R.; Liu, J.; Zuo, P.; Li, Q.; Hu, M.; Meng, L.; Wang, K.; Smilgies, D. M.; Zhao, G.; Xu, H.; Yang, Z.; Amassian, A.; Li, J. *et al.* Multi-inch single-crystalline perovskite membrane for high-detectivity flexible photosensors. *Nature Communications* **2018**, *9*, 5309.

6. Tu, Y.; Xu, Y.; Li, J.; Hao, Q.; Liu, X.; Qi, D.; Bao, C.; He, T.; Gao, F.; Zhang, W. Ultrathin Single-Crystalline 2D Perovskite Photoconductor for High-Performance Narrowband and Wide Linear Dynamic Range Photodetection. *Small* **2020**, *16*, 1–8.

7. Zhang, Y.; Liu, Y.; Xu, Z.; Yang, Z.; Liu, S. 2D Perovskite Single Crystals with Suppressed Ion Migration for High-Performance Planar-Type Photodetectors. *Small* **2020**, *16*, 2003145.

8. Liu, Y.; Ye, H.; Zhang, Y.; Zhao, K.; Yang, Z.; Yuan, Y.; Wu, H.; Zhao, G.; Yang, Z.; Tang, J.; Xu, Z.; Liu, S. F. Surface-Tension-Controlled Crystallization for High-Quality 2D Perovskite Single Crystals for Ultrahigh Photodetection. *Matter* **2019**, *1*, 465–480.

9. Xu, Z.; Li, Y.; Liu, X.; Ji, C.; Chen, H.; Li, L.; Han, S.; Hong, M.; Luo, J.; Sun, Z. Highly Sensitive and Ultrafast Responding Array Photodetector Based on a Newly Tailored 2D Lead Iodide Perovskite Crystal. *Advanced Optical Materials* **2019**, *7*, 1900308.

10. Zhao, Y.; Qiu, Y.; Gao, H.; Feng, J.; Chen, G.; Jiang, L.; Wu, Y. Layered-Perovskite Nanowires with Long-Range Orientational Order for Ultrasensitive Photodetectors. *Advanced Materials* **2020**, *32*, 1905298.

11. Bao, C.; Chen, Z.; Fang, Y.; Wei, H.; Deng, Y.; Xiao, X.; Li, L.; Huang, J. Low-Noise and Large-Linear-Dynamic-Range Photodetectors Based on Hybrid-Perovskite Thin-Single-Crystals. *Advanced Materials* **2017**, *29*, 1703209.

12. Jing, H.; Peng, R.; Ma, R. M.; He, J.; Zhou, Y.; Yang, Z.; Li, C. Y.; Liu, Y.; Guo, X.; Zhu, Y.; Wang, D.; Su, J.; Sun, C.; Bao, W.; Wang, M. Flexible Ultrathin Single-Crystalline Perovskite Photodetector. *Nano Letters* **2020**, *20*, 7144–7151.

13. Li, C.; Wang, H.; Wang, F.; Li, T.; Xu, M.; Wang, H.; Wang, Z.; Zhan, X.; Hu, W.; Shen, L. Ultrafast and broadband photodetectors based on a perovskite/organic bulk heterojunction for large-dynamic-range imaging. *Light: Science & Applications* **2020**, *9*, 31.

14. Tong, G.; Geng, X.; Yu, Y.; Yu, L.; Xu, J.; Jiang, Y.; Sheng, Y.; Shi, Y.; Chen, K. Rapid, stable and self-powered perovskite detectors via a fast chemical vapor deposition process. *RSC Advances* **2017**, *7*, 18224–18230.

15. Feng, X.; Tan, M.; Li, M.; Wei, H.; Yang, B. Polyhydroxy Ester Stabilized Perovskite for Low Noise and Large Linear Dynamic Range of Self-Powered Photodetectors. *Nano Letters* **2021**, *21*, 1500–1507.

16. Ji, Z.; Cen, G.; Su, C.; Liu, Y.; Zhao, Z.; Zhao, C.; Mai, W. All-Inorganic Perovskite Photodetectors with Ultrabroad Linear Dynamic Range for Weak-Light Imaging Applications. *Advanced Optical Materials* **2020**, *8*, 1–9.

17. Bao, C.; Yang, J.; Bai, S.; Xu, W.; Yan, Z.; Xu, Q.; Liu, J.; Zhang, W.; Gao, F. High Performance and Stable All-Inorganic Metal Halide Perovskite-Based Photodetectors for Optical Communication Applications. *Advanced Materials* **2018**, *30*, 1803422.

18. Zhou, H.; Zeng, J.; Song, Z.; Grice, C. R.; Chen, C.; Song, Z.; Zhao, D.; Wang, H.; Yan, Y. Self-Powered All-Inorganic Perovskite Microcrystal Photodetectors with High Detectivity. *Journal of Physical Chemistry Letters* **2018**, *9*, 2043–2048.

19. Tang, X.; Zhou, H.; Pan, X.; Liu, R.; Wu, D.; Wang, H. All-Inorganic Halide Perovskite Alloy Nanowire Network Photodetectors with High Performance. *ACS Applied Materials and Interfaces* **2020**, *12*, 4843–4848.

20. Liu, H.; Yang, P.; Song, H.; Guo, Y.; Zhan, S.; Huang, H.; Wang, H.; Tao, B.; Mu, Q.; Xu, J.; Li, D.; Chen, Y. Generalized weighted ratio method for accurate turbidity measurement over a wide range. *Optics Express* **2015**, *23*, 32703.

21. RS Hydro: INW Turbo Sensor. `https://www.rshydro.co.uk/water-quality-monitoring-equipment/water-quality-testing-equipment/water-quality-sensors/turbidity-sensors-probes/inw-turbo-turbidity-sensor/`, (Accessed: 17.10.2023).

22. Vernier: Turbidity Sensor. `https://www.vernier.com/product/turbidity-sensor/`, (Accessed: 17.10.2023).

23. Aqualabo: Turbidity Sensor. `https://en.aqualabo.fr/userfiles/doc/Datasheet%20Turbidity%20digital%20sensor%20NTU.pdf`, (Accessed: 17.10.2023).

24. Valeport: Hyperion Turbidity Sensor. https://www.valeport.co.uk/content/uploads/2021/10/Valeport-Hyperion-Turbidity-Datasheet-October-23.pdf, (Accessed: 17.10.2023).

25. Real Tech: Real Turbidity Sensor. https://realtechwater.com/products/single-parameter-sensors/turbidity-sensor/, (Accessed: 17.10.2023).

26. Luimstra, V. M.; Schuurmans, J. M.; Verschoor, A. M.; Hellingwerf, K. J.; Huisman, J.; Matthijs, H. C. Blue light reduces photosynthetic efficiency of cyanobacteria through an imbalance between photosystems I and II. *Photosynthesis Research* **2018**, *138*, 177–189.

27. Agusti, S.; Phlips, E. J. Light absorption by cyanobacteria: Implications of the colonial growth form. *Limnology and Oceanography* **1992**, *37*, 434–441.
